# Sublobar resection is associated with improved outcomes over radiotherapy in the management of high-risk elderly patients with Stage I non-small cell lung cancer: a systematic review and meta-analysis

**DOI:** 10.18632/oncotarget.14010

**Published:** 2016-12-17

**Authors:** Huan-Huan Wang, Chun-Ze Zhang, Bai-Lin Zhang, Jie Chen, Xian-Liang Zeng, Lei Deng, Mao-Bin Meng

**Affiliations:** ^1^ Department of Radiation Oncology, Tianjin's Clinical Research Center for Cancer and Key Laboratory of Cancer Prevention and Therapy, Tianjin Medical University Cancer Institute and Hospital, National Clinical Research Center for Cancer, Tianjin 300060, China; ^2^ Department of Colorectal Surgery, Tianjin Union Medical Center, Tianjin 300121, China; ^3^ Department of Thoracic Cancer and Huaxi Student Society of Oncology Research, West China Hospital, West China School of Medicine, Sichuan University, Sichuan Province 610041, China

**Keywords:** non-small cell lung cancer, radiotherapy, sublobar resection, overall survival, pattern of failure

## Abstract

**Background and Aim:**

A matched-pair comparison was performed to compare the efficacy and safety of sublobar resection versus radiotherapy for high-risk elderly patients with Stage I non-small cell lung cancer (NSCLC).

**Patients and Methods:**

We searched the Cochrane Library, MEDLINE, CENTRAL, EMBASE and manual searches. The meta-analysis was performed to compare overall survival, pattern of failure, and toxicity among the homogeneous studies. Subdivided analyses were also performed.

**Results:**

Sixteen studies containing 11540 patients were included in the meta-analysis. Among these studies, 9 were propensity-score matched (PSM) cohort studies, and 7 were cohort studies. Sublobar resection, compared with radiotherapy (either conventional fraction radiation therapy or stereotactic body radiation therapy), significantly improved the overall survival regardless in both PSM and non-PSM analyses (all *p* < 0.05). However, the difference in the pattern of failure and toxicity were not significant (all *p* > 0.05).

**Conclusions:**

Sublobar resection was associated with improved outcomes in high-risk elderly patients with Stage I NSCLC, which supports the need to compare both treatments in large prospective, randomized, controlled clinical trials.

## INTRODUCTION

Anatomic resection (lobectomy or pneumonectomy) with systematic lymph node dissection or sampling is the mainstay of therapy in early-stage non-small cell lung cancer (NSCLC) [1–2]. However, more than half of patients are not candidates for the standard surgical procedure, most often because they are elderly or have pulmonary dysfunction, poor performance status, or comorbidities [3]. In these compromised patients, various treatment strategies are available including observation, conventional fractionated radiation therapy (CFRT), stereotactic body radiation therapy (SBRT), radiofrequency ablation, or sublobar resection (SLR, i.e. wedge resection or segmentectomy) with or without systematic lymph node dissection [2, 4–5].

The majority of compromised patients with early-stage NSCLC who are not eligible for lobectomy should be offered an alternative local treatment, as more than half of patients with inoperable cancer who do not undergo treatment will die of disease [6–8]. Radiofrequency ablation is an emerging modality for the treatment of inoperable stage I NSCLC; however, data for radiofrequency ablation are less mature because of small sample size, undefined adaptive tumor volumes, and shorter follow-ups [11–13]. CFRT used for the local treatment of early-Stage NSCLC patients with poor pulmonary reserve has been used reluctantly due to poor local control and lung toxicity [9–10]. Therefore, the two most optimal modalities for compromised Stage I NSCLC patients appear to be SLR and SBRT according to the European Organization for Research and Treatment of Cancer and the International Society of Geriatric Oncology [14].

Both SLR and SBRT are associated with excellent local control rates, and they also have shown the capacity to maintain pulmonary function post-procedure [14]. However, the question remains as to which intervention is the best treatment for compromised patients who cannot tolerate lobectomy. To resolve this issue, an intergroup randomized trial (RTOG 1021/ACOSOG Z4099) comparing SLR with SBRT in high-risk patients with Stage I NSCLC was initiated [15]. Unfortunately, it was closed in May 2013 due to slow patient enrollment. The aim of this study was to conduct a meta-analysis to evaluate the efficacy and safety of treatments for high-risk elderly patients with Stage I NSCLC, with comparison between two treatment groups: SLR versus radiotherapy (either CFRT or SBRT). It is anticipated that this meta-analysis will provide evidence-based information for clinical practice.

## RESULTS

### Search results

The systematic literature search identified a total of 3792 relevant references. After careful reading of the titles and abstracts, 3769 references were excluded because the objective did not satisfy the inclusion criteria. A total of 23 studies were retrieved for further assessment. Of these, five studies were excluded because of an inability to obtain the SLR data [16–20] and two studies were excluded because of duplication [21–22]. Ultimately, 16 studies (11,540 patients) [23–38] met our inclusion criteria.

### Common characteristics

A majority of the included studies were conducted in the USA, except for two studies [23, 38]. All the patients were diagnosed with Stage I NSCLC, and the median age was 66 years and older. A PSM comparison was performed in 9 studies, and a total of 10,870 matched patients were included [25, 27–28, 31–35, 37]. Five studies evaluated the efficacy and safety of SLR versus CFRT for these patients [23–27]; 10 studies were designed to compare SLR versus SBRT [28–37]; and one study evaluated the efficacy and safety of SLR versus CFRT and SBRT [38], as shown in Table 1.

**Table 1:** Main characteristics of all the included studies

Study			Main characteristics			Overall survival				Patterns of failure (No. of patients)					
Author (Year)	Country	Design	Arms	No. of Patients	Age	1-year	2-year	3-year	5-year	LF	RF	LDF	RDF	DF	LRF
SLR or WR versus CFRT															
Yano T (1995) ^23^	Japan	Cohort	CFRT SLR	18 17	74.5 68.5	72.8% 95.7%	63.4% 95.7%	14.4% 61.5%	14.4% 55%	4 3	NA	NA	NA	NA	NA
Ghosh S (2003) ^24^	UK	Cohort	CHART WR	19 47	76.9 76.6	80% 98%	80% 90%	68.4% 86.4%	39% 74%	5 9	NA	6 13	NA	1 4	NA
Yendamuri S (2007) ^25^	USA	Cohort (Matched pairs)	3D-CRT WR	34 34	72 72	86.2% 91.7%	80% 82.2%	58.3% 65.4%	38.7% 41.5%	NA	NA	NA	NA	NA	NA
Hsie M (2009) ^26^	USA	Cohort	3D-CRT WR	39 45	73 70.5	88.3% 98.4%	67.1% 79.6%	55% 62.7%	31.2% 56%	5/38 7/40	4/38 5/40	12/38 16/40	11/38 14/40	7/38 9/40	9/38 12/40
Fernandez FG (2012) ^27^	USA	Cohort (Matched pairs)	3D-CRT SLR	319 319	77 75.5	77.4% 87.5%	52% 71.6%	41% 52%	20% 41%	NA	NA	NA	NA	NA	NA
SLR or WR versus SBRT															
Forquer JA (2009) ^28^	USA	Cohort (Matched pairs)	SBRT SLR	19 19	66 67	NA	NA	NA	NA	0 2	4 3	1 1	0 1	1 0	4 5
Grills IS (2010) ^29^	USA	Cohort	SBRT WR	58 69	78 74	81.7% 98.2%	74.5% 92%	56% 81.3%	56% 52.4%	3 14	3 13	14 29	14 28	11 15	6 27
Parashar B (2010) ^30^	USA	Cohort	SBRT WR ± BT	25 22	77.5 71.5	NA	NA	NA	NA	1 1	NA	6 3	NA	5 2	NA
Varlotto J (2013) ^31^	USA	Cohort (Matched pairs)	SBRT WR	137 48	73.3 67.5	68.2% 100%	50.8% 94.1%	42.3% 86.3%	31.7% 86.3%	NA	NA	NA	NA	21 4	15 6
Shirvani SM (2012) ^32^	USA	Cohort (Matched pairs)	SBRT SLR	112 112	75 75	80.2% 81.9%	61.4% 63.7%	56.7% 60.5%	NA	NA	NA	NA	NA	NA	NA
Port JL (2014) ^33^	USA	Cohort (Matched pairs)	SBRT WR ± BT	23 76	76 72	NA	NA	75% 87%	NA	1 1	2 1	5 3	6 3	4 2	3 2
Matsuo Y (2014) ^34^	Japan	Cohort (Matched pairs)	SBRT SLR	53 53	76 76	94.5% 94.2%	80% 83.5%	61.2% 72.9%	40.4% 55.6%	NA	NA	NA	NA	NA	NA
Puri V (2015) ^35^	USA	Cohort (Matched pairs)	SBRT SLR	4555 4555	73.8 73.7	87.1% 89%	62.4% 76.3%	47% 61.7%	26.9% 43.1%	NA	NA	NA	NA	NA	NA
Parashar B (2015) ^36^	USA	Cohort	SBRT WR	97 123	77 77	96.2% 100%	91.5% 100%	91.5% 97.7%	89.6% 97.7%	4 10	NA	22 32	NA	18 22	NA
Paul S (2016) ^37^	USA	Cohort (Matched pairs)	SBRT SLR	201 201	77.6 75.6	90.5% 92.5%	69% 81.3%	57.4% 67.2%	27.8% 62%	NA	NA	NA	NA	NA	NA
Safi S (2015) ^38 †^	Germany	Cohort	CFRT or SBRT SLR	49 42	73.5 69.6	94% 93%	69% 85%	18% 66%	NA	17 4	NA	NA	NA	NA	NA

^†^ Among 49 patients receiving RT, 28 and 21 patients have received CFRT and SBRT, respectively.

*Abbreviations*: CHART: continuous hyperfractionated accelerated radiotherapy; CFRT: conventional fractionated radiotherapy; SLR: sublobar resection; LF: local failure; RF: regional failure; LDF: local and distant failure; RDF: regional and distant failure; DF: distant failure; LRF: locoregional failure; WR: wedge resection; BT: brachytherapy; mths: months; 3D-CRT: three-dimensional conformal radiotherapy; NA: not reported.

For patients undergoing SLR, the main operation was thoracotomy or video-assisted thoracoscopic surgery (VATS) with or without systematic lymph node dissection or sampling. The dosage range in the CFRT group was 45.0–90.3 Gy in 22–40 fractions, and that in the SBRT group was 30–66 Gy in 2–8 fractions. The treatment characteristics of the included studies are listed in Table 2.

**Table 2:** Technical features of SLR, CFRT, and SBRT for treatment of high-risk elderly patients with stage I NSCLC

Study	SLR or WR		CFRT or SBRT				
Author (Year)	Resection type	MLN dissection	Prescription dose (Gy)	No. of fractions	Does per fraction (Gy)	BED_10_ (Gy)	Isodose line (%)
SLR or WR versus CFRT							
Yano T (1995)^23^	Thoracotomy	NA	51.2, 60, 70, 80	30–40	1.6 or 2.0	59.39, 72, 84, 96	95%
Ghosh S (2003)^24^	Thoracotomy	Cervical medianstinoscopy	54	36	1.5	62.1	95%
Yendamuri S (2007)^25^	VATS or Thoracotomy	MLN dissection or sampling	66 (45–90.3)	22	3	85.8	95%
Hsie M (2009)^26^	VATS or Thoracotomy	None	70 (60–75)	28	2.5 (2.0–4.11)	87.5	95%
Fernandez FG (2012)^27^	Thoracotomy	± MLN sampling	NA	NA	NA	NA	95%
SLR or WR versus SBRT							
Forquer JA (2009)^28^	Thoracotomy	± MLN sampling	60 or 66	3	20 or 22	180 or 211.2	80
Grills IS (2010)^29^	VATS or Thoracotomy	± Mediastinoscopy or MLN dissection	48 or 60	4 or 5	12	105.6 or 132	80 (60–90)
Parashar B (2010)^30^	Thoracotomy	None	30–60	2–4	10–15	60–150	100
Varlotto J (2013)^31^	Thoracotomy	± MLN sampling	48–60	3–5	12, 20	67.2, 78, 90	NA
Shirvani SM (2012)^32^	Thoracotomy	± MLN sampling	NA	NA	NA	NA	NA
Port JL (2014)^33^	VATS or Thoracotomy	± MLN sampling	48 (30–60)	4 (3–5)	12 (6–20)	NA	100
Matsuo Y (2014)^34^	VATS or Thoracotomy	± MLN sampling	48, 56, 60	4, 4, 8	12, 14, 7.5	105.6, 134.4, 105	NA
Puri V (2015)^35^	VATS or Thoracotomy	± MLN sampling	53.83 ± 6.78	NA	NA	NA	NA
Parashar B (2015)^36^	Thoracotomy	Mediastinoscopy	48 (30–60)	4 (3–5)	12 (10–12)	105.6	100
Paul S (2016)^37^	VATS or Thoracotomy	± MLN sampling	NA	NA	NA	NA	NA
Safi S (2015)^38†^	VATS or Thoracotomy	MLN dissection or sampling	CFRT: 66 SBRT: 45 (median)	CFRT: 33 SBRT: 3 (median)	CFRT: 2 SBRT: 18 (median)	CFRT: 79.2 SBRT: NA	95% NA

^†^Among 49 patients receiving RT, 28 and 21 patients have received CFRT and SBRT, respectively.

Abbreviations: CFRT: conventional fractionated radiotherapy; SLR: sublobar resection; MLN: mediastinal lymph node; Gy: Gray; BED: biologically equivalent dose; VATS: video-assisted thoracoscopic surgery; SBRT: stereotactic body radiation oncology; NA: not reported.

### Survival outcome and patterns of failure

The included studies presented Kaplan-Meier curves, numbers of events, and 1-, 2-, 3-, and 5-year OS rates. In these studies, 5782 patients were treated with SLR, 429 patients were treated with CFRT, and 5329 patients were treated with SBRT. We compared the ORs for OS and patterns of failure between SLR and radiotherapy either CFRT or SBRT.

SLR, compared with radiotherapy either CFRT or SBRT, led to significantly better the 1-, 2-, 3-, and 5-year OS rates regardless of whether the analysis was PSM or non-PSM (all *p* < 0.05). When we recalculated the results after excluding one study because had considerable weight [47], we found that SLR, compared with SBRT, was associated with significantly better 3- and 5-year OS rates in the PSM analyses (all *p* < 0.05). However, the difference in the pattern of failure and toxicity were not significant (all *p* > 0.05) (Tables 3 and 4).

**Table 3:** SLR versus CFRT or SBRT for high-risk elderly stage I NSCLC: a meta-analysis of OS

End point	No. of studies	No. of patients	OR	95% CI	Significance	Publication bias	Heterogeneity
SLR versus CFRT							
1-year survival							
All studies combined	5	891	2.30	1.57–3.37	0.0001	0.46	0.54
PSM analysis	2	706	2.01	1.34–3.03	0.001		0.87
None PSM analysis	3	185	6.20	1.88–20.50	0.003		1.00
2-year survival							
All studies combined	5	891	2.11	1.59–2.81	0.0001	0.31	0.11
PSM analysis	2	706	2.10	1.54–2.87	0.0001		0.05
None PSM analysis	3	185	2.18	1.08–4.40	0.03		0.15
3-year survival							
All studies combined	5	891	1.54	1.18–2.01	0.002	0.81	0.28
PSM analysis	2	706	1.53	1.14–2.06	0.005		0.71
None PSM analysis	3	185	1.59	0.87–2.91	0.14		0.09
5-year survival							
All studies combined	5	891	2.73	2.02–3.69	0.0001	0.81	0.67
PSM analysis	2	706	2.80	1.99–3.94	0.0001		0.86
None PSM analysis	3	185	2.50	1.32–4.72	0.05		0.32
SLR versus SBRT							
1-year survival							
All studies combined	8	10465	1.64	1.02–2.64	0.04	1.00	0.05
PSM analysis	5	10027	1.44	1.32–1.57	0.0001		0.16
PSM analysis excluding reference 50	4	917	1.63	0.65–4.09	0.29		0.04
None PSM analysis	3	438	4.62	1.66–12.86	0.003		0.06
2-year survival							
All studies combined	8	10465	1.90	1.20–3.02	0.006	1.00	0.0001
PSM analysis	5	10027	1.95	1.26–3.02	0.003		0.003
PSM analysis excluding reference 50	4	917	2.15	0.98–4.75	0.06		0.002
None PSM analysis	3	438	9.01	3.88–20.93	0.0001		0.08
3-year survival							
All studies combined	9	10564	2.91	1.94–4.38	0.0001	0.75	0.0001
PSM analysis	6	10126	2.29	1.48–3.55	0.0001		0.0001
PSM analysis excluding reference 50	5	1016	2.17	1.21–3.87	0.009		0.007
None PSM analysis	3	438	5.63	3.22–9.86	0.0001		0.05
5-year survival							
All studies combined	6	10588	2.97	1.51–5.83	0.002	1.00	0.0001
PSM analysis	4	9803	3.74	1.92–7.26	0.0001		0.003
PSM analysis excluding reference 50	3	693	3.55	1.06–11.94	0.04		0.002
None PSM analysis	2	347	1.83	0.37–9.13	0.46		0.03

Abbreviations: CFRT: conventional fractionated radiotherapy; PSM: propensity-score matched; SBRT: stereotactic body radiation therapy; SLR: sublobar resection; OR: Odds ratio; CI: confidence interval.

**Table 4:** SLR versus CFRT or SBRT for high-risk elderly Stage I NSCLC: a meta-analysis of pattern of failures

End point	No. of studies	No. of patients	OR	95% CI	Significance	Publication bias	Heterogeneity
SLR versus CFRT							
LF	3	185	1.09	0.50–2.36	0.84	1.00	0.69
RL	1	84	0.82	0.20–3.33	0.79		
DF	2	150	0.74	0.27–1.98	0.54		0.84
LRF	1	84	0.72	0.26–1.98	0.53		
SLR versus SBRT							
LF							
All studies combined	6	622	0.83	0.23–3.02	0.78	0.46	0.008
PSM analysis	2	137	0.68	0.11–4.31	0.68		0.16
None PSM analysis	4	485	0.83	0.17–3.96	0.81	0.73	0.003
RF							
All studies combined	3	264	1.08	0,66–7.03	0.94	0.30	0.03
PSM analysis	2	137	2.29	0.60–8.81	0.23		0.28
None PSM analysis	1	127	0.23	0.06–0.87	**0.03**		
DF							
All studies combined	6	716	1.36	0.88–2.11	0.16	0.09	0.25
PSM analysis	3	322	2.79	1.10–7.06	**0.03**		0.44
None PSM analysis	3	394	1.05	0.63–1.76	0.84		0.55
LRF							
All studies combined	4	449	0.77	0.22–2.47	0.69	0.73	0.007
PSM analysis	3	322	1.12	0.53–2.40	0.76		0.18
None PSM analysis	1	127	0.18	0.07–0.48	**0.001**		

Abbreviations: CFRT: conventional fractionated radiotherapy; PSM: propensity-score matched; SBRT: stereotactic body radiation therapy; SLR: sublobar resection; OR: Odds ratio; CI: confidence interval; LF: local failure; RF: regional failure; DF: distant failure; LRF: locoregional failure.

### Heterogeneity analysis and publication bias

**Figure 1:**
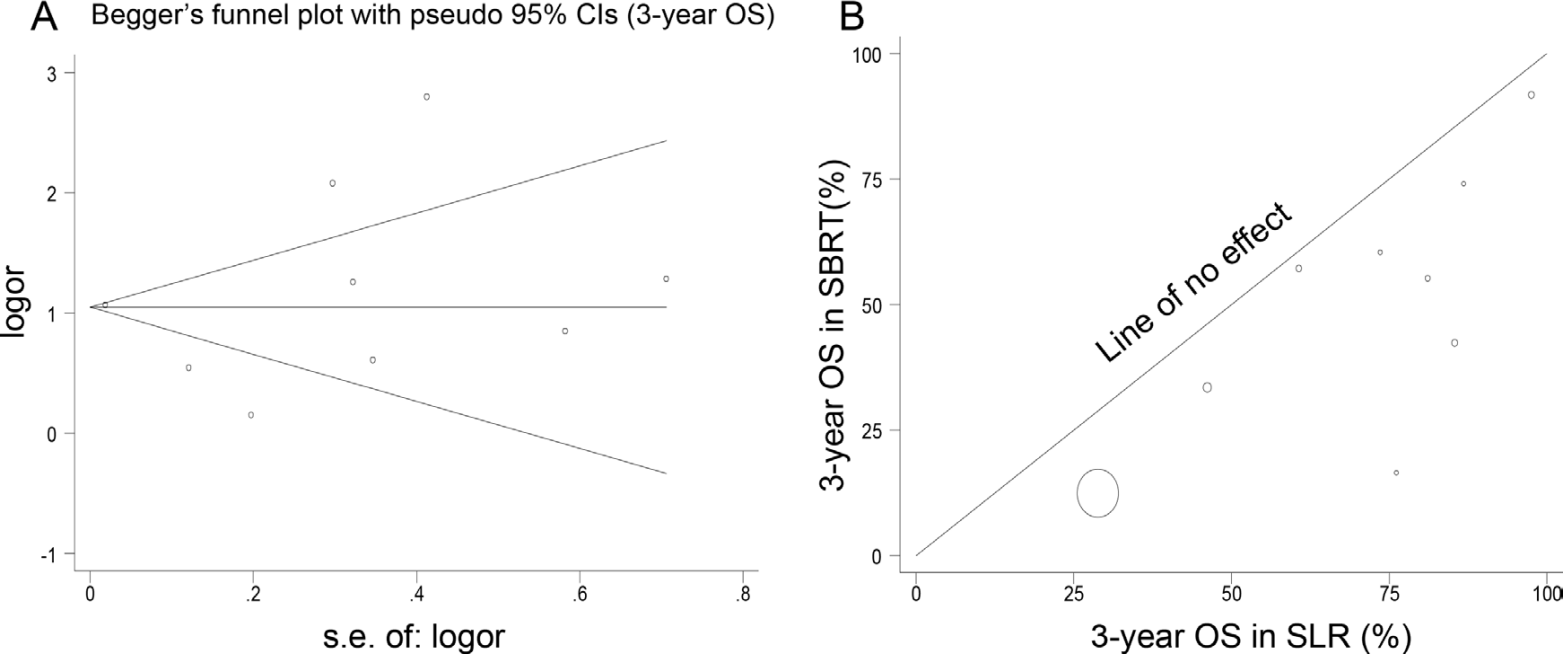
Analyses of publication bias and heterogeneity (**A**) The funnel plot appears symmetric, and there was no evidence of publication bias for SLR versus SBRT using the Bagg rank correlation method (*p* = 0.75 for 3-year OS). The horizontal line in the funnel plot indicates the fixed-effects summary estimate, and the sloping lines indicate the expected 95% CIs for a given standard error, assuming no heterogeneity between studies. (**B**) L’Abbé plot showing the 3-year OS rates, comparing the effect size in SBRT and SLR.

There was evidence of heterogeneity for OS and pattern of failure (Tables 3 and 4). L’Abbé plots of 3-year OS of SLR versus SBRT showed evidence of heterogeneity (Figure 1A). However, a review of funnel plots could not rule out the potential for publication bias for either analysis. Publication bias was not evident when the Begg rank correlation method and Egger's Weighted regression method (*p* = 0.75 for 3-year OS and *p* = 0.46 for local failure) were used for SLR versus SBRT (Figure 1B).

### Sensitivity analysis

A sensitivity analysis was performed to explore the influence of study quality on the effect size. In the primary analysis, outcomes of 3-year OS and local failure for SLR versus SBRT were applied in a random-effects model. In terms of 3-year OS, when we recalculated the sensitivity analysis after application of a fixed-effects model, we found that the overall estimates were virtually identical and the CIs were similar between the sensitivity analysis (OR = 2.80; 95% CI 2.53–3.09; *p* = 0.001) and the meta-analysis (OR = 2.91; 95% CI 1.94–4.38; *p* = 0.0001). In addition, we found that the OR and 95% CI for local failure were also similar (OR = 0.87; 95% CI 0.50–1.51; *p* = 0.62) and (OR = 0.83; 95% CI 0.23–3.02; *p* = 0.78).

## DISCUSSION

To our knowledge, this study represents the only available quantitative assessment of published data on SLR versus radiotherapy either CFRT or SBRT for high-risk elderly patients with Stage I NSCLC. The present study revealed that SLR was associated with a better OS compared with radiotherapy either CFRT or SBRT. Although such studies have some limitations, together they contain credible evidence that the administration of each treatment modality is worthy of additional study. It is hoped that this will help to better define the roles of these therapies for high-risk elderly patients with Stage I NSCLC.

It was noted that SLR was included segmentectomy and wedge resection in this study. Theoretically, segmentecomy is considered superior ontologically than wedge resection because it provides a larger parenchymal margin and an increased nodal yield [39–40]. For example, Ezer N et al. assessed the efficacy of SBRT versus segmentecomy or wedge resection separately using SEER database, and found that SBRT treated patients had significantly worse OS and lung cancer-specific OS compared with patients treated with segmentecomy. Nevertheless, OS and lung cancer-specific OS after wedge resection and SBRT were not significantly different [41]. However, some studies have shown that lobectomy and segmentectomy for small clinical Stage I NSCLC are equivalent, whereas wedge resection showed inferior outcomes [42–44]. Consistent with our results and these findings, further studies are warranted for SBRT versus SLR stratified by segmentecomy or wedge resection.

Since SBRT does not intentionally treat lymphatic nodal basins, nodal staging is of critical importance. In this study, the majority of included studies reported that PET-CT was used for patients who received SBRT. PET-CT staging of NSCLC has been shown to have a sensitivity of 85% and specificity of 90% [45]. Low rates of isolated nodal failure for NSCLC after PET-CT staging of 2.3%–10% are reported in the literature [46–47]. In practice, SLR and SBRT can be used to address the primary tumor, but do not treat the mediastinal or intralobar lymph nodes. As PET-CT and other imaging modalities continue to more accurately assess the status of the lymph nodes in NSCLC, the relevance of highly targeted treatments, such as SBRT, for stage I NSCLC patients will further increase.

Treatment-related toxicity is an important factor in selecting and appropriate therapy. Seven studies [24, 27–28, 31–32, 35, 37] in this study reported adverse events. SLR, CFRT, and SBRT have specific complications. CFRT and SBRT cause toxicity to the normal structures that surround the tumor, resulting in esophagitis, pneumonitis, hemoptysis, and chest wall pain, but no deaths were attributed to CFRT or SBRT. SLR can affect patients’ quality of life in different ways such as further impairment of pulmonary function and chronic pain such as arrhythmia, myocardial infarction, pneumonia, thoracic empyema, and severe lung hemorrhage. In addition, it was noted that whether elderly patients face an increased risk of complications following SBRT for early-Stage NSCLC, as has been reported following surgery. Mancini et al. recently demonstrated that elderly patients undergoing SBRT for early-Stage NSCLC appear to have similar risk of toxicity and rate of efficacy as in younger patients [48].

We believe PSM analysis was performed to reduce selection bias between patients with SLR and SBRT. In this study, PSM accounted for factors of age, gender, tumor stage, pathology, lung function, ECOG performance score, race, comorbidity index i.e. Charlson Comorbidity Index [27, 29, 31–34, 36, 38], and tumor grade, etc. Importantly, we found that compared with SBRT, SLR significant improved OS in the PSM analysis; however, the pattern of failure after SLR was similar to that after SBRT (Tables 3 and 4).

Some limitations of the present study must be acknowledged. First, there was no randomization, and the comparison was subject to bias. Second, some studies did not perform PSM meta-analysis may be influence the outcome. Third, the radiographic definitions of failure were probably different between the patients treated with SLR and those treated with radiotherapy. After SLR, there would be no visible tumor left behind, whereas after radiotherapy, a visible tumor frequently persists and local control is defined as no growth. In addition, some differences between SLR and radiotherapy were observed. Histological confirmation of NSCLC was performed before treatment for all cases receiving radiotherapy, but not SLR. However, patients underwent surgery have pathological staging, but not radiotherapy.

The choice of treatment for high-risk elderly patients with early-Stage NCSLC should be made based on several variables such as patient characteristics, tumor characteristics, and local practice. The development of a personalized treatment model to determine the best treatment for high-risk elderly patients with early-Stage NCSLC based on several such characteristics might be the next step in the treatment of these patients. It will be beneficial to define the impact of each treatment modality on patient care in terms of cost, survival, and improvement in quality of life and to determine the optimal combination therapy for effective palliation and cure of high-risk elderly patients with early-Stage NSCLC.

In conclusion, the findings of this meta-analysis suggest that SLR was associated with better overall survival compared over radiotherapy either CFRT or SBRT in the management of high-risk elderly patients with Stage I NSCLC. Considering the strength of the evidence, additional randomized controlled trials are needed before each treatment modality can be recommended routinely.

## MATERIALS AND METHODS

### Criteria for inclusion

Acceptable publications met the following criteria: 1) patients with Stage I NSCLC; 2) age > 65 years; 3) a treatment group receiving SLR with or without systematic lymph node dissection or sampling and a control group receiving radiotherapy either CFRT or SBRT; and 4) reported data for overall survival (OS) or patterns of failure for calculation of the odds ratios (ORs) and 95% confidence intervals (CIs).

### Exclusion criteria

Studies were excluded if they did not meet the criteria above and 1) involved animal studies or *in vitro* studies; 2) did not represent primary research (reviews, editorials, commentaries, case reports, and letters to the editor); 3) represented duplicate publications of other studies previously identified in our systematic evaluation; or 4) investigated toxicity only, without OS or pattern of failures, were also excluded.

### Search strategy

Retrieval of trials was performed through the Cochrane Library, MEDLINE, CENTRAL, and EMBASE. The search was designed to initially find all trials involving the terms: “non-small cell lung cancer” or “non-small cell lung carcinoma” or “carcinoma, non-small cell lung” and “sublobar resection” or “SLR” or “wedge resection” or “segmentectomy” or “segmental resection” and “radiation therapy” or “radiotherapy” or “RT” or “stereotactic body radiation therapy” or “stereotactic radiotherapy” or “stereotactic body radiotherapy” or “stereotactic body radiosurgery” or “stereotactic ablative body radiotherapy” or “stereotactic ablative radiotherapy” or “SBRT” or “SABR” (and multiple synonyms for each term). We also manually searched the general reviews on NSCLC and references from published clinical trials. The search results were downloaded to a reference database and screened further.

### Outcome measurements

Outcome measurements of these trials comprised: 1) OS; 2) patterns of failure; and 3) adverse events. Survival included 1-, 2-, 3-, and 5-year survival rates, which were extracted from actual numbers reported in the trials or derived from the survival curves. The lacked key information for calculation with methods developed by Parma et al. [49], Williamson et al. [50], and Tierney et al. [51].

Patterns of failure included local failure, regional failure, distant metastasis, and locoregional failure. Local failure included primary tumor recurrence and recurrence in the involved lobe; regional failure was defined as tumor recurrence in the ipsilateral uninvolved lobe or ipsilateral hilar and mediastinal nodes; and distant metastasis was defined as any disease in contralateral nodes or distant sites. Primary tumor recurrence was diagnosed on the basis of histologic confirmation or enlargement of the local tumor on CT that continued for at least 6 months. Positron emission tomography (PET)-CT was considered when primary tumor recurrence was highly suspected [52–53].

### Review methods

### Data extraction

Three reviewers independently selected the trials and performed the data extraction. Discrepancies were resolved by discussion among reviewers. Information lacking in the original publications was supplemented through correspondence with the original principal investigator. Finally, the following information was extracted from each included trial: 1) the characteristics of the study; 2) the number of patients allocated and patient characteristics; 3) the interventional measures used (CFRT, SBRT, and SLR); and 4) outcomes such as OS, patterns of failure, and adverse events.

### Statistical methods

We performed the meta-analysis by pooling CFRT or SBRT and SLR data for an overall analysis regardless of study design. To determine the influence of the PSM analysis on the conclusions of the meta-analysis, subgroup analysis was conducted [54]. Pooled ORs were presented as standard plots with 95% CIs. All *p-values* were two-sided, and *p* < 0.05 was considered statistically significant.

In addition, inter-trial heterogeneity in treatment effect was evaluated using both the Q statistic test and a visual display in a L’Abbé plot [55]. Second, a random-effects model was employed using the DerSimonian and Laird (DL) method [56] to calculate 95% CIs, resulting in wider intervals and, thus, a more conservative estimate of treatment effects compared with a fixed-effects model using the Mentel-Haenszel (MH) method. For trials in which the constructed 2 × 2 tables contained cells with zero events, a standard correction factor of 0.5 was added to each cell. Third, we applied two different statistical models (a fixed-effects model and a random-effects model) to perform the sensitivity analysis in accordance with the recommendations from the Cochrane Collaboration and the Quality of Reporting of Meta-analysis guidelines. Finally, Begg and Mazumdar's proposed adjusted rank correlation test [57] and Egger's linear regression approach [58] were used to measure publication bias (*p* < 0.05 was considered representative of statistically significant publication bias), which was shown as a funnel plot. Analysis was performed using the statistical software Intercooled Stata version 8.2 for Windows (Stata Corporation, College Station, TX, USA).
